# Pennsylvania Hospital

**Published:** 1839-03-30

**Authors:** 


### CLINICAL LECTURE.

### PENNSYLVANIA HOSPITAL.

### LITHOTOMY.

*Wednesday*, *March* 20*th*.—Dr. Randolph entered the lecture room, and commenced as follows:—

Gentlemen,—We have at this time two little patients in the house afflicted with stone in the bladder. The eldest child is ten years of age, and the youngest two. I shall bring before you presently the child two years old, and I propose to extract the stone by means of the operation of *lithotomy*. I shall bring the child, ten years of age, before you, most probably, on this day week, and I shall, if practicable, perform on it the operation of *lithotripsy*. It would seem that the little patient upon whom we propose to operate to-day, has been labouring under the complaint for about twelve months. We are informed by his parents that they noticed, when he was one year old, that the passage of his urine was attended with some difficulty. Since that time, the symptoms attending this painful disorder have been gradually increasing in violence, and have now assumed a most distressing character.

I presume you are all acquainted with the symptoms attendant upon the existence of a calculus in the bladder. In the commencement of the disorder the patient experiences a frequent desire to pass his urine, and an inability to hold it for any length of time; he also feels an uneasy sensation at the mouth of the urethra and glans penis, in consequence of which he has an uncontrollable desire to compress the penis, and to stretch and elongate the prepuce. As the stone increases in size, the discharge of urine is mostly attended with excruciating pain, particularly after voiding the last drops of it; this is owing to the bladder contracting forcibly upon the stone. Sometimes a good deal of mucous matter, and, not unfrequently, some blood, is discharged along with the urine. Very often the flow of water is suddenly stopped by the stone falling upon the mouth of the urethra; this induces the patient to strain violently for a few seconds; after a time, it will recommence flowing, or else it passes out by drops. Occasionally, the contents of the bowels are discharged involuntarily during the efforts to empty the bladder. The patient is also subject to paroxysms, or fits of the stone, as they have been called, at which periods his sufferings are much increased. Notwithstanding these symptoms which I have just described are quite sufficient to indicate clearly, in most instances, the existence of a calculus in the bladder, still, in order to determine this point positively, it is necessary to introduce a sound, and detect the stone by means of this instrument. Having ascertained the true nature of the complaint, the only means in our power of relieving the patient consists in freeing him from the stone by some kind of operation. You are aware that two methods of operating may be resorted to for the purpose of extracting a stone from the bladder; one method consists in cutting open some part of the bladder, and extracting the stone by means of a pair of forceps; this constitutes the operation of *lithotomy*. Another method consists in introducing suitable instruments into the bladder, and crushing the stone into pieces, which are then discharged through the urethra along with the urine; this constitutes the operation of *lithotripsy*. I need not inform you, that within the last few years, surgeons have succeeded most happily and successfully in very many instances in effecting a complete cure of this painful malady, by means of this method. Now, gentlemen, I profess, myself, to be an entire convert to the superiority of the operation of *lithotripsy* over *lithotomy*. I contend that the former operation has many advantages over the latter; that it is much more exempt from the hazard of immediate danger, and that it is less liable to be followed by unpleasant consequences. Amongst the objections which may be enumerated against the operation of lithotomy, I may notice the danger of the patient dying soon after the operation, from convulsions, or from a sudden and great prostration of his vital powers from the shock communicated to his system by the operation; the danger of fatal hæmorrhage ensuing from the division of vessels out of sight and out of reach; the danger of death occurring a short time subsequently to the operation from inflammation of the bladder, or general peritoneal inflammation; besides which, the liability of incontinence of urine resulting from the operation, or a fistula in perineo. It is proper, however, that I should inform you, that these objections apply more particularly, and with greater force, to the operation of lithotomy performed upon an adult, than upon a child. Children, in general, recover more speedily and readily from the effects of operations than grown persons, or such as are somewhat advanced in years; besides which, the operation of lithotomy upon a child is a very different affair from the same operation upon an adult; it is much more readily performed—the depth of the perineum is less considerable—the membranous part of the urethra is more readily exposed—the cavity of the bladder is more within reach, and, consequently, the stone is more readily extracted. Notwithstanding all this, children occasionally die from the effects of the operation of lithotomy, even in cases when it has been performed in the most skilful manner possible.

My reasons for resorting to the operation of lithotomy upon the present occasion, are—the extreme youth of the patient, and the difficulty of getting him to submit to the necessary repetitions of the operation of lithotripsy; besides which, we are not provided with instruments adapted to the small size of his urethra.

Since engaging in the operation of *lithotripsy*, I have had under my care *twenty-four* cases of stone occurring in adults; in *nineteen* of these cases I performed the operation of lithotripsy. I considered this method entirely adapted to three of the other patients; they, however, disappeared from under my notice, and, I suppose, fell into the hands of other surgeons. Upon the remaining two patients I was obliged to perform the operation of *lithotomy*. So that out of *twenty-four* cases of stone occurring in adults, I have met with but *two* in which the operation of lithotripsy has not been admissible. One of these patients had an ulcer in the urethra, and, consequently, could not bear the repeated introduction of the instruments; and the other was an old man, who may be said to have had scarcely any bladder at all. He had laboured under the complaint for many years; and the coats of his bladder were so much thickened, that its cavity was not larger than a walnut, and was almost entirely filled up by the calculus, a projecting extremity of which had been forced into the prostatic portion of the urethra. During this period, I have cut four children for stone, and have performed lithotripsy upon two others in this house. One of these was eleven years of age, and the other four. I have not performed the operation upon a younger child than this. In the American Journal of the Medical Sciences, six cases were reported about a year since by Prof. N. R.

Smith, in which he performed lithotripsy successfully. Four of these cases were children—one under two years of age, another under three years, and the two others were seven years old.

I shall not occupy your time with an account of the several methods of performing the operation of lithotomy. That which we have generally performed in this city is called the lateral operation, and it is done in this manner:

A grooved staff is first introduced into the bladder,—next the hands and feet are secured together by means of strong muslin bands, to prevent the struggles of the patient from interfering with the operation. Then an incision is made in the perineum, commencing just behind the raphe of the scrotum, and terminating midway between the anus and the tuberosity of the ischium; this incision divides the skin and transversales perinei muscles, together with the fascia. The next step is to dissect down to the membranous part of the urethra, and divide it, laying bare the groove of the staff. Some surgeons use a bistoury at this stage of the operation, but I prefer employing the same scalpel with which the first incisions are made. By turning the back of the knife to the prostate gland, the membrane can be readily divided forwards towards the bulb. The nail of the forefinger of the left hand is next introduced into the groove of the staff, and then the beak of the gorget inserted by the side of it; it is now moved backward and forward a few times, to ascertain that no portion of membrane intervenes between the beak of the instrument and the groove. You then pass the gorget along the groove dividing the prostate gland and neck of the bladder laterally, after which the stone must be extracted. Dr. Physick's gorget is preferred by many surgeons, as the blade can be separated from the beak, and a much keener edge given to it than in the old instrument, which was made of one piece. If any important vessels are divided, and bleed much, they must be secured. The patient should then be placed in bed upon his left side. Previous to performing the operation, it is advisable to keep the patient still for a short time, and to allay the irritability of his system and bladder; and on the day preceding the operation, his bowels should be emptied by means of some mild cathartic medicine, such as magnesia or castor oil. It is proper also to administer to the patient, about two hours before the operation, some preparation of opium; I generally give to an adult fifty or sixty drops of laudanum, or, instead of this, two or three grains of opium, thrown into the rectum by injection,—and to a child, a dose in proportion to its age.

[The patient was now brought in, and Dr. R. performed the operation as mentioned above, except that it was not found necessary to use the forceps to extract the stone. By means of a finger introduced into the rectum, the stone was instantly thrown out through the wound. The operation occupied about one minute.]

*March 30th.*—The patient has not experienced a single unpleasant symptom since its performance.

